# Lack of phenotypic effect of triallelic variation in *SPATA7* in a family with Leber congenital amaurosis resulting from *CRB1* mutations

**Published:** 2011-12-16

**Authors:** Lin Li, Xueshan Xiao, Shiqiang Li, Xiaodong Jiao, J. Fielding Hejtmancik, Qingjiong Zhang

**Affiliations:** 1 State Key Laboratory of Ophthalmology, Zhongshan Ophthalmic Center, Sun Yat-Sen University, Guangzhou, China; 2 Ophthalmic Genetics and Visual Function Branch, National Eye Institute, National Institutes of Health, Bethesda, MD

## Abstract

**Purpose:**

To identify the causative gene for autosomal recessive Leber congenital amaurosis (LCA) in a Chinese family.

**Methods:**

One Chinese LCA family was identified and an ophthalmologic examination was performed. The genetic defects were analyzed simultaneously by a genome-wide linkage scan with 382 polymorphic microsatellite markers, as well as by comprehensive mutational screening of 15 genes known to associate with LCA on the genomic DNA of this family.

**Results:**

Suggestive linkages were found in 13 chromosomal regions, of which only one harbored a known causative gene, crumbs homolog 1 (*CRB1*), on chromosome 1. Sanger sequencing of *CRB1* identified two novel heterozygous mutations, c.3221T>C (p.L1074S) and c.2677–2A>C. In addition, a novel missense heterozygous mutation, c.938C>A (p.A313D), in spermatogenesis associated 7 (*SPATA7*), was detected in the proband after screening of the other 14 LCA causative genes. All three affected individuals of the family had compound heterozygous *CRB1* mutations, and one of the three (the proband) had an additional mutation in *SPATA7*. The unaffected mother had the heterozygous c.3221T>C mutation in *CRB1* and the heterozygous c.938C>A mutation in *SPATA7*. The unaffected father could not be tested, but presumably had the heterozygous c.2677–2A>C mutation in *CRB1*. The proband, with triallelic mutations in *CRB1* and *SPATA7*, had a phenotype similar to other two affected brothers, suggesting the additional mutant allele in *SPATA7* might not contribute to the disease. Similarly, the mother, with digenic mutations in *CRB1* and *SPATA7*, had normal vision and fundi, suggesting the digenic mutations in these two genes might not cause disease.

**Conclusions:**

Digenic and triallelic mutations of *CRB1* and *SPATA7* were detected in a family with LCA. Our results imply that *CRB1* and *SPATA7* may not interact with each other directly. This emphasizes that care should be taken in invoking a mutation–disease association for digenic and triallelic mutations.

## Introduction

Leber congenital amaurosis (LCA, OMIM 204000) is an extreme and highly heterogeneous form of retinal dystrophy, characterized by severe visual loss at or near birth, Franceschetti's oculodigital sign, searching or roving nystagmus, and pigmentary retinopathy [1,2]. Visual acuity is rarely better than 20/400 [3], and fundus changes are extremely variable, ranging from normal appearance to obvious pigmentary retinopathy similar to retinitis pigmentosa. Electroretinogram (ERG) recordings are usually flat lines or severely abnormal [2]. The prevalence of LCA is around one to two per 80,000 live births, accounting for approximately 20% of cases of inherited blindness among children in institutes for the blind and more than 5% of all congenital retinopathies. At present, 18 LCA loci have been mapped, in which 17 causative genes have been identified: guanylate cyclase 2D (*GUCY2D*) [4], crumbs homolog 1 (*CRB1*) [5], retinal pigment epithelium-specific protein 65 kDa (*RPE65*) [6], retinitis pigmentosa GTPase regulator interacting protein 1 (*RPGRIP1*) [7], aryl hydrocarbon receptor interacting protein-like 1 (*AIPL1*) [8], Leber congenital amaurosis 5 (*LCA5*) [9], cone-rod homeobox (*CRX*) [10], lecithin retinol acyltransferase (*LRAT*) [11], tubby like Protein 1(*TULP1*) [12], retinol dehydrogenase 12 (RDH12) [13], centrosomal Protein 290 kDa (*CEP290*) [14], retinal degeneration 3 (*RD3*) [15], spermatogenesis associated 7 (*SPATA7*) [16], (inosine 5′-monophosphate [IMP]) dehydrogenase 1 (*IMPDH1*) [17], orthodenticle homeobox 2 (*OTX2*) [18], IQ motif containing B1 (*IQCB1*) [19], and calcium binding protein 4 (*CABP4*) [20]. Although LCA was mostly thought to be transmitted as a recessive and dominant trait [2], some LCA cases show a triallelic or digenic inheritance [21,22].

Here, we report an autosomal recessive LCA family with three affected members from Guangdong Province, China. After a genome-wide linkage scan and comprehensive mutational screening, two novel mutations in *CRB1* and one novel mutation in *SPATA7* were identified in the family, demonstrating digenic mutations in an unaffected individual, triallelic mutations in one affected individual, and compound heterozygous mutations in two affected individuals with a phenotype indistinguishable from their sibling with triallelic mutations.

## Methods

### Leber congenital amaurosis family

Family 83002 with LCA was collected from the Pediatric and Genetics Clinic of the Zhongshan Ophthalmic Center, Sun Yat-sen University, Guangzhou, China. They were of the Chinese Han ethnicity and lived in Yangjiang of Guangdong Province, China. The pedigree and clinical phenotype suggested autosomal recessive inheritance of the disease in this family. Written informed consent conforming to the tenets of the Declaration of Helsinki and following the Guidance of Sample Collection of Human Genetic Diseases (863-Plan) by the Ministry of Public Health of China was obtained from the participating individuals or their guardians before the study. Three affected siblings and the unaffected mother of the family participated in this linkage study. Genomic DNA was prepared from leukocytes of peripheral venous blood.

### Genotype analysis and linkage analysis

A genome-wide linkage scan was performed with 382 highly polymorphic fluorescent markers from the ABI PRISM Linkage Mapping Set MD-10 (Applied Biosystems, Foster City, CA). Multiplex PCR was performed in a GeneAmp PCR System 9700 thermocycler (Applied Biosystems). Briefly, each reaction was performed in a 5 μl mixture containing 20 ng genomic DNA. Initial denaturation was performed for 5 min at 95 °C, followed by 10 cycles of 15 s at 95 °C, 15 s at 55 °C, and 30 s at 72 °C, and then 20 cycles of 15 s at 89 °C, 15 s at 55 °C, and 30 s at 72 °C. The final extension was performed for 10 min at 72 °C. PCR products for a subset of markers from each subject were pooled and mixed with HD-400 size standards (Applied Biosystems). Different alleles were separated using an ABI 3130 DNA analyzer (Applied Biosystems) and then assigned using GeneMapper (version 4.0; Applied Biosystems). Two-point linkage analyses were performed using the MLINK from the LINKAGE program package [23,24]. Maximum logarithm of odds (LOD) scores were calculated using ILINK. Autosomal recessive LCA was analyzed as a fully penetrant trait with an affected allele frequency of 0.0001. The marker order and distances between the markers were obtained from the Genethon database.

### LCA candidate gene screening

All candidate gene in regions with a suggestive linkage were analyzed by Sanger sequencing. Simultaneously, 15 genes known to associate with LCA were analyzed by Sanger sequencing, as previously described [25], including *GUCY2D*, *CRB1*, *RPE65*, *RPGRIP1*, *AIPL1*, *LCA5*, *CRX*, *LRAT*, *TULP1*, *RDH12*, *CEP290*, *RD3*, *SPATA7*, *IMPDH1*, and *OTX2*. Briefly, PCR amplifications were performed in 10 μl reactions containing 40 ng genomic DNA. Touchdown PCR amplification consisted of a denaturizing step at 96 °C for 5 min, followed by decreasing the annealing temperature from an initial 64 °C by 0.5 °C every second cycle for 15 cycles, an annealing temperature of 57 °C for 21 cycles, and incubation at 72 °C for 10 min. Sequencing results were assembled using an ABI PRISM 3130 automated sequencer (Applied Biosystems) and analyzed with Seqman software (DNAStar Lasergene 8, Madison, WI) and a mutation surveyor (SoftGenetics, State College, PA). A missense mutation was predicted to be damaging by the position-specific independent-counts algorithm of Polyphen [26], by a Sorting Intolerant From Tolerant (SIFT) score (which distinguishes tolerated variants from those that are not tolerated) equal to or less than 0.05, and by being absent in 192 ethnically matched control chromosomes. The splicing changes were predicted by Automated Splice Site Analyses.

## Results

### Mutation detection

LOD scores greater than 1.0 were obtained in 13 chromosomal regions (data not shown), of which only one harbored a gene known to cause LCA, *CRB1* on chromosome 1, which gave a LOD score of 1.18, near the theoretical maximum for this family (Table 1).

**Table 1:** Two-Point lod scores of family 83002 for makers on 1p.

	**Position**		**Lod score at theta=**						
**Markers**	cM*	**Mb#**	**0**	**0.01**	**0.05**	**0.1**	**0.2**	**0.3**	**0.4**
D1S218	196.5	174.5	-inf	−0.81	−0.18	0.02	0.12	0.09	0.03
D1S238	206.7	188.1	1	0.98	0.87	0.74	0.48	0.24	0.06
*CRB1*		197.2	1.2	1.18	1.07	0.93	0.64	0.34	0.1
D1S413	216.5	198.6	0.49	0.47	0.43	0.37	0.24	0.12	0.03
D1S249	225.1	205.7	0.49	0.47	0.43	0.37	0.24	0.12	0.03
D1S425	235.3	212.1	0.49	0.47	0.43	0.37	0.24	0.12	0.03
D1S213	246.2	223.8	-inf	−0.97	−0.34	−0.12	0.02	0.03	0.01

• Genethon • # Homo genome (Build 37.2) Chr1 Primary_Assembly.

**Figure 1:**
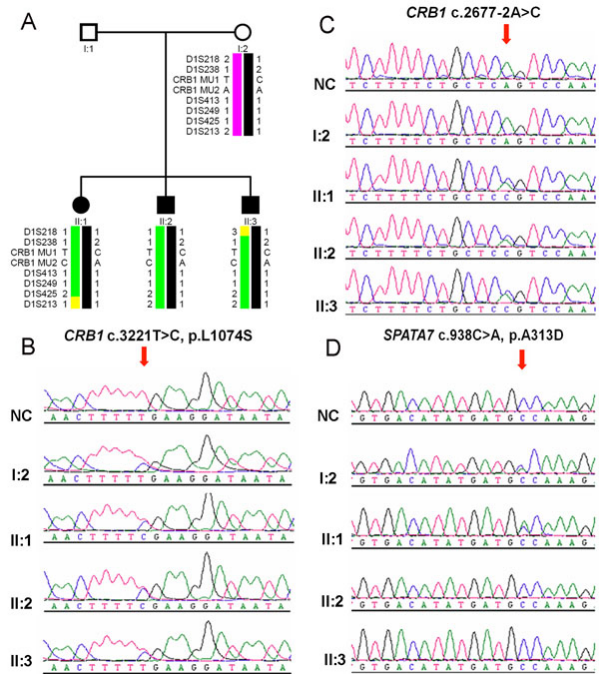
Pedigree and haplotypes: sequence changes of family 83002. (The DNA sample for I:1 was not available.) **A**: Haplotypes of the *CRB1* region of family 83002 showing the *CRB1* c.3221T>C and c.2677–2A>C mutations and surrounding microsatellite markers included in Table 1. The risk haplotype is shown in black and green. **B**, **C**: Electropherograms demonstrate sequences in the regions with the *CRB1* c.3221T>C and c.2677–2A>C mutations in individuals I:2, II:1, II:2, II:3, and a normal control. **D**: Electropherograms show the c.938C>A mutation in *SPATA7* of individuals I:2 and II:1.

Sanger sequencing of *CRB1* identified two novel heterozygous mutations, c.3221T>C (p.L1074S) and c.2677–2A>C, in the proband (II:1). Simultaneous comprehensive mutation screening in the remaining 14 of the 15 known LCA genes detected a single additional novel missense heterozygous mutation, c.938C>A (p.A313D), in *SPATA7* in the proband (Figure 1). No mutation was detected in *GUCY2D*, *RPE65*, *RPGRIP1*, *AIPL1*, *LCA5*, *CRX*, *LRAT*, *TULP1*, *RDH12*, *CEP290*, *RD3*, *IMPDH1*, or *OTX2*.

The other two affected patients (II:2 and II:3) in this family had only the two compound heterozygous *CRB1* mutations without the c.938C>A mutation in *SPATA7* (Figure 1). The unaffected mother had the heterozygous c.3221T>C mutation in *CRB1* and the heterozygous c.938C>A mutation in *SPATA7*. By implication, the unaffected father presumably had the heterozygous c.2677–2A>C mutation in *CRB1*.

**Figure 2:**
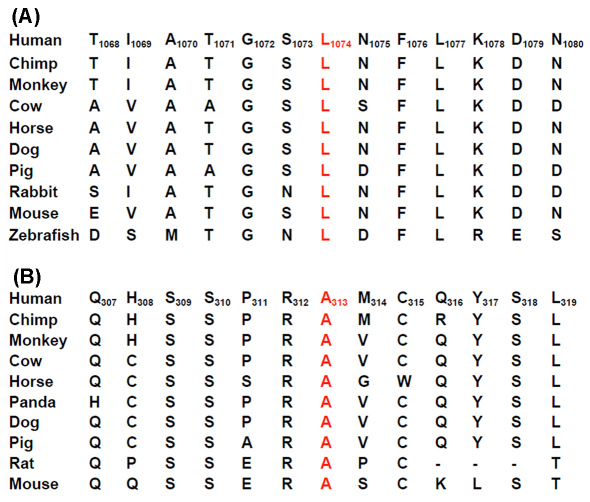
Protein sequence alignment. **A**: *CRB1 L1047* amino acid(red) in ten species ranging from humans to zebrafish. L1047 is conserved in all species from *Homo sapiens* to *Danio rerio* as ascertained by a bioinformatic search of NCBI-Blast by means of human CRB1 DNA and protein and MegAlign (DNASTAR Lasergene, Madison, WI). And this may indicate functional or structural significance in L1047. **B**: *SPATA7* A313 amino acid(red) in ten species ranging from humans to mice. A313 is relatively conserved among mammal.

None of the three mutations were detected in the 192 chromosomes of the normal controls. Of the three mutations, the c.2677–2A>C mutation in *CRB1* was predicted to abolish the splicing site, altering the invariant 2A residue in intron 7. The other two mutations, c.3221T>C (p.L1074S) in CRB1 and c.938C>A (p.A313D) in *SPATA7,* were predicted to be damaged by SIFT or probably damaging by Polyphen (Table 2). The L1074 in *CRB1* and the A313 in *SPATA7* were relatively well conserved residues (Figure 2).

**Table 2:** Mutations in the 15 genes detected in 83002 family with LCA and in 192 chromosomes of controls.

**Gene**	**Nucleotide change**	**Amino acid change**	**Conservation**	**Computational prediction**			**Remark**	**Occurance in controls**	**Report**
				Blosum62*	**PolyPhen or Splice Site**	**SIFT**			
*CRB1*	c.3221T>C	L1074S	Yes	6	probably damaging	damaging	Pathogenic	0/192	This study
	c.2677–2A>C	N/A	N/A	N/A	splicing site abolished	N/A	Pathogenic	0/192	This study
*SPATA7*	c.938C>A	A313D	Yes	6	probably damaging	damaging	Pathogenic	0/192	This study

• Difference between the scores. Except for mutations in CRB1 and *SPATA1*, no other changes were detected in 13 other LCA genes, including *GUCY2D, RPE65, RPGRIP1, AIPL1, LCA5, CRX, LRAT, TULP1, RDH12, CEP290, RD3, IMPDH1* and *OTX2*.

### Clinical phenotype

**Figure 3:**
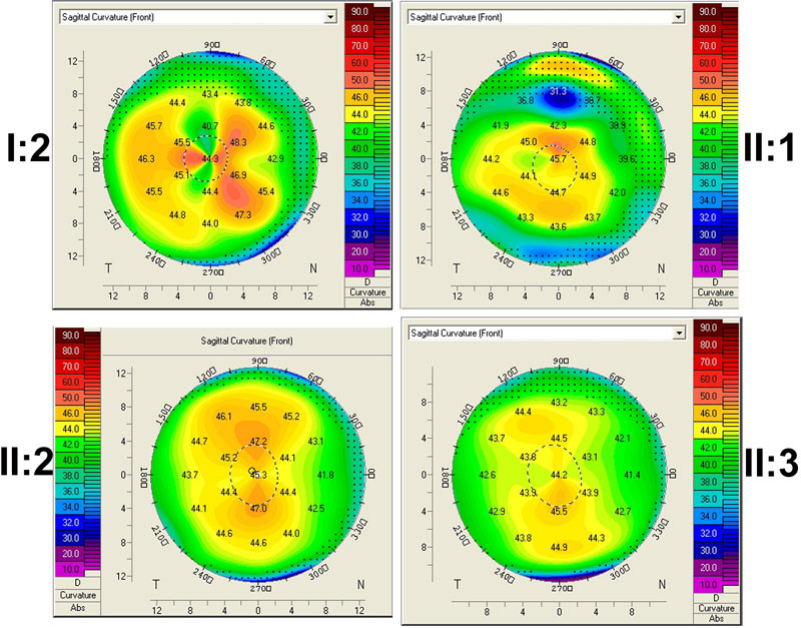
Sagittal curvature of the cornea. Only the right eye of the four members of the family are shown, since the sagittal curvatures of both eyes in each individual were similar. The number of color scale presents the degree of the curvature of cornea. The corneal diameters and corneal curvatures of the three patients(II:1, II:2 and II:3) were similar with unaffected mother (I:2) and within normal range.

**Figure 4:**
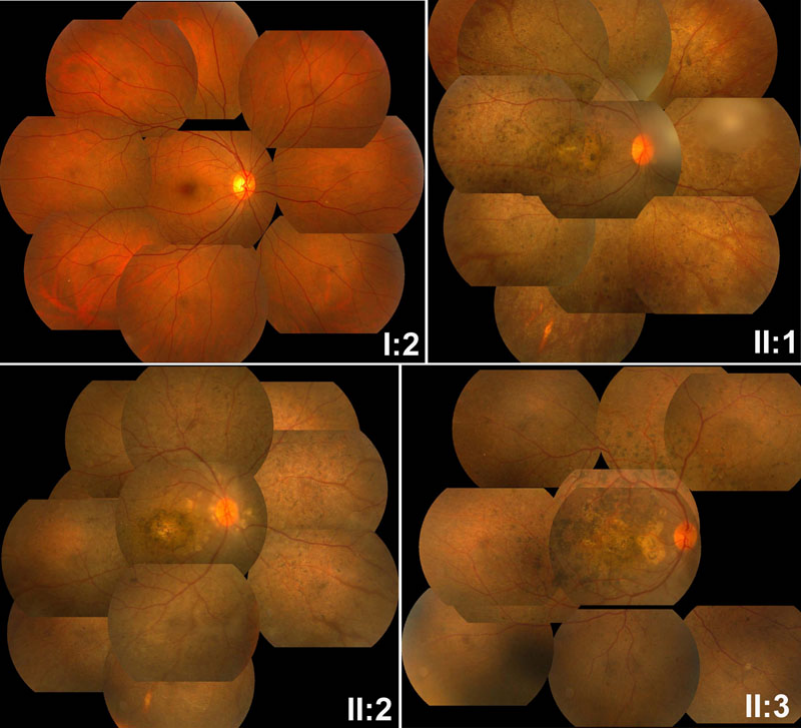
Fundus photos of the four members of the family. The mother (I:2), with digenic mutations, had a normal fundus appearance. All three patients (II:1, II:2, and II:3) from the family had similar fundus changes, including waxy, pale optic discs, artery attenuation, generalized carpetlike retinal degeneration, macular atrophy, nummular pigmentation at the posterior pole, and irregular pigmentation and white dots in the midperipheral region. The fundus changes in the proband (II:1) with triallelic mutations in *CRB1* and *SPATA7* were similar to the those of the other two patients (II:2 and II:3) with compound heterozygous *CRB1* mutations.

**Figure 5:**
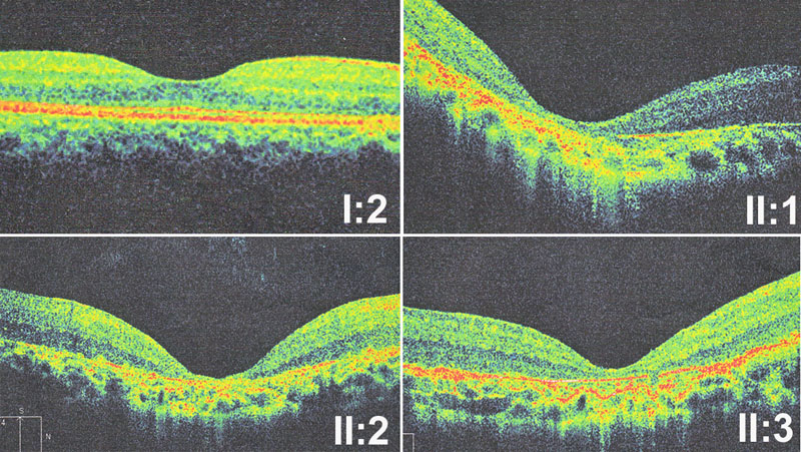
Optical coherence tomography of three patients shows thinning of the retina and loss of photoreceptor layers compared with the mother (I:2) who has normal fundus. Patient II:1 had similar changes, compared to patients II:2 and II:3. It indicated the affected member II:1 who carried a third mutant allele in a second gene may not have more severe phenotypes than other affected members in the family.

Clinical information of the family members is listed in Table 3. All three patients from the family were found to have poor vision and nystagmus in the first few months after birth. The ERGs of rod and cone responses were unrecordable. The patients in this family first visited our clinic when they were preschool children. Their visual acuity, fundus changes, and ERG recordings were similar during follow-up visits for over ten years. For patient II:1, the right eye’s visual acuity was 0.04 and the left eye’s was 0.05. For patient II:2, the right eye’s visual acuity was 0.02 and the left eye was finger counting. For patient II:3, the right eye’s visual acuity was 0.06 and the left eye’s was 0.03. All three patients had hyperopia correlated with short axial length, which suggested microphthalmia (Table 3). The corneal diameters and corneal curvatures of the three patients were similar and within normal range (Figure 3). The fundus changes in all three patients were similar, including the waxy paleness of optic disc, artery attenuation, generalized carpetlike retinal degeneration, macular atrophy, nummular pigmentation at the posterior pole, and irregular pigmentation and white dots at the midperipheral region (Figure 4). Optical coherence tomography showed thinning of the retina and loss of photoreceptor layers (Figure 5). Overall, the proband (patient II:1) with triallelic mutations in *CRB1* and *SPATA7* had a phenotype similar to the other two affected brothers’, as well as better visual acuity than that of most LCA patients with other CRB1 mutations or with mutations in other LCA genes [25], suggesting that the additional mutant allele in *SPATA7* might not contribute to the disease. The mother with digenic mutations in *CRB1* and *SPATA7* had normal vision, a normal fundus appearance, and a normal visual field (data not shown), suggesting that the digenic mutations in these two genes might not be causative.

**Table 3:** Clinical Information of the individuals in the 83002 Family.

**ID**	**Variations**		**Age (year)**	**First symptom**	**Age at onset**	**Axial length (right/left, mm)**	**Visual acuity (right/left eye)**	**ERG rod and cone responses**
	***CRB1***	***SPATA7***						
I:2	c.3221T>C	c.938C>A	44	No	No	21.26/21.23	1.0; 1.0	Normal
II:1	c.3221T>C;	c.938C>A	22	PV, NYS*	FFMAB*	19.52/19.53	0.04; 0.05	Undetectable
	c.2677–2A>C							
II:2	c.3221T>C;	No	20	PV, NYS	FFMAB	19.31/19.95	0.02; FC	Undetectable
	c.2677–2A>C							
II:3	c.3221T>C;	No	18	PV, NYS	FFMAB	19.53/19.93	0.06; 0.03	Undetectable
	c.2677–2A>C							

*Note: ERG: Flash ERG. The undetectable records indicated both cone and rod cell degenerated in three patients. PV=poor vision (decreased visual acuity); NYS=nystagmus; FFMAB=first few months after birth.

## Discussion

Digenic and triallelic mutations have been identified as causes of hereditary retinal degeneration [21,27-31]. LCA is the earliest-occurring and most severe inherited retinal degeneration, and is mostly inherited as an autosomal recessive trait [2]. Mutations in at least 17 genes have been identified as being responsible for LCA (RetNet). It was expected that digenic and triallelic mutations would be detected in patients with LCA, and we did identify digenic and triallelic mutations in our previous study [25]. It has been suggested in previous studies that affected members in families that carry a third allele in a second gene have more severe phenotypes than other affected members [21,32]. However, further studies are expected to reveal whether digenic mutations are causative in general, and whether triallelic mutations usually cause a severe phenotype.

Here, we identified digenic mutations in *CRB1* and *SPATA7* in a healthy mother and triallelic mutations in a proband with LCA after comprehensive genetic study of a Chinese family with LCA. The fundus pictures of the three patients showed typical features of *CRB1*-related LCA: nummular pigmentation and macular atrophy. The proband with triallelic mutations in *CRB1* and *SPATA7* had a phenotype similar to the other two affected brothers, suggesting that the additional mutant allele in *SPATA7* might not contribute to the disease. The mother with digenic mutations in *CRB1* and *SPATA7* had a normal ocular fundus, suggesting that digenic mutations in these two genes might not be causative. This suggests that digenic mutations may not always result in clinical consequences, even if they are pathogenic mutations, and that they may, on their own, cause disease in the context of a homozygous or compound heterozygous status. Triallelic mutations may not necessarily cause a severe phenotype, even they can cause the same disease by themselves. Our results imply that *CRB1* and *SPATA7* might not interact with each other directly.

Digenic or triallelic mutations are difficult to detect if these genes are analyzed independently. However, such mutations will probably be seen more frequently, given the widespread application of genotyping microarrays, next-generation sequencing, and exome sequencing. Cosegregation analyses based on family members will still be an important tool in validating the clinical significance of digenic or triallelic mutations. Great care should be taken in making mutation-disease associations between digenic and triallelic mutations before there is firm phenotypic evidence to support such associations, especially in clinical gene diagnoses.
